# Comparing the techniques and outcomes of laparoscopic transverse colectomy to laparoscopic hemicolectomy in mid-transverse colon cancer resection

**DOI:** 10.3389/fsurg.2022.1012947

**Published:** 2023-01-06

**Authors:** Rui Sun, Guannan Zhang, Xiyu Sun, Beizhan Niu, Jiaolin Zhou, Lin Cong, Huizhong Qiu, Guole Lin, Bin Wu, Yi Xiao

**Affiliations:** Division of Colorectal Surgery, Department of General Surgery, Peking Union Medical College Hospital, Chinese Academy of Medical Sciences, Peking Union Medical College, Beijing, China

**Keywords:** mid-transverse colon cancer, transverse colectomy, right hemicolectomy, left colectomy, bowel function

## Abstract

**Introduction:**

The mid-transverse colon cancer is relatively uncommon in all colon cancers and the optimal surgical approach of mid-transverse colon cancer remains debatable.

**Aim and Objectives:**

Our study aimed to depict the techniques and outcomes of laparoscopic transverse colectomy in one single clinical center and compare this surgical approach to traditional laparoscopic right hemicolectomy and laparoscopic left hemicolectomy.

**Method:**

This was a retrospective cohort study of patients with mid-transverse colon cancer in one single clinical center from February 2012 to October 2020. The enrolled patients were divided into two groups undergoing laparoscopic transverse colectomy and laparoscopic right/left hemicolectomy, respectively. The intraoperative, postoperative complications, oncological outcomes and functional outcomes were compared between the two groups. The primary endpoint was disease free survival (DFS).

**Results:**

The study enrolled 70 patients with 40 patients undergoing laparoscopic transverse colectomy and 30 patients undergoing laparoscopic hemicolectomy. The intraoperative accidental hemorrhage and multiple organ resection occurred similarly in the two groups. In transverse colectomy, caudal-to-cephalic approach was likely to harvest more lymph nodes although require more operation time than cephalic-to-caudal approach (23.1 ± 14.3 vs. 13.4 ± 5.4 lymph nodes, *P* = 0.004; 184.3 ± 37.1 min vs. 146.3 ± 44.4 min, *P* = 0.012). The laparoscopic transverse colectomy was marginally associated with lower incidence of overall postoperative complications and shorter postoperative hospital stay although without statistical significance (8(20.0%) vs. 12(40.0%), *P* = 0.067; 7(5–12) vs. 7(5–18), *P* = 0.060). The 3-year DFS showed no significant difference (3-year DFS 89.7% in transverse colectomy vs. 89.9% in hemicolectomy, *P* = 0.688) between the two groups. The alternating consistency of defecation occurred significantly less after laparoscopic transverse colectomy than laparoscopic hemicolectomy (15(51.7%) vs. 20(80.0%), *P* = 0.030).

**Conclusion:**

The laparoscopic transverse colectomy is technically feasible with satisfactory oncological and functional outcomes for mid-transverse colon cancer. Performing the caudal-to-cephalic approach might be more advantageous in lymphadenectomy.

## Introduction

Colon cancer is the third most common type of non-skin cancer and is associated with significant morbidity and mortality attributed to its aggressive metastasis. Neoplastic cells can arise along the entire course of the colon; however, transverse colon cancer remains a relatively rare subset of colon cancer which accounts for approximately 10% (1) of all colorectal cancer. Surgery is the crucial procedure to treat colon cancer. Because of its scarcity and variability of surgical approaches, transverse colon cancer has been excluded from large randomized controlled trials that aimed to compare outcomes between laparoscopic colectomy and open surgery (2–5). With the location usually between the left and right branches of the middle colic artery (MCA), surgical management of mid-transverse colon cancer can be shifted from localized transverse colectomy to entended right or left hemicolectomy. Transverse colectomy is referred to the segmental resection of transverse colon with ligation and resection of MCA at its root. While hemicolectomy is referred to regionally resection of right-sided or left-sided colon with ligation and resection of at least two main feeding arteries.

The more advantageous surgical approach for mid-transverse colon cancer remains debatable. Several retrospective studies (6–8) have reported that transverse colectomy did not alter recurrence patterns, and showed equivalent long-term outcomes to hemicolectomy. However, hemicolectomy remains the most prevalently used treatment method for mid-transverse colon cancer (6, 9). An Italian national retrospective study found that transverse colectomy is associated with a higher incidence of anastomotic leak and compromised oncological outcomes (9).

Previous studies reported that injury or resection of the autonomic nerve plexus and also the length of resected bowel can theoretically increase the risk of diarrhea and affect bowel function after laparoscopic right hemicolectomy (10–12). Thus, transverse colectomy may confer an advantage by preserving the terminal ileum, ascending colon, and ileocecal valve, as well as lowering the risk of autonomic nerve plexus injury.

The standardized procedure of mid-transverse colon cancer remains undefined, while transverse colectomy also indicates technical challenges and flexible decisions such as how to make tension-free anastomosis and how to accomplish thorough lymphadenectomy. Our study aimed to discuss the detailed techniques of laparoscopic transverse colectomy and to compare the outcomes of laparoscopic transverse colectomy to those of laparoscopic hemicolectomy.

## Materials and methods

### Patients

This single-center retrospective cohort study complied with the Declarations of Helsinki and was approved by the Research Ethics Committee of the Peking Union Medical College Hospital. The recruitment criteria were as follows: patients diagnosed with mid-transverse colon adenocarcinoma without distant metastasis and underwent laparoscopic radical surgery between February 2012 and October 2020. Mid-transverse colon cancer was defined as colon cancer located in the middle one-third of the transverse colon. Patients with other malignant diseases, distant metastasis, multiple primary colorectal cancer, and those undergoing palliative surgery and total and subtotal colorectal resection were excluded.

### Surgical procedure

Enrolled patients underwent laparoscopic transverse colectomy or laparoscopic hemicolectomy at the surgeons' discretion. All laparoscopic surgeries were performed using a three-dimensional (3D) imaging system with the camera port site placed between the pubic symphysis and umbilicus using a 10 mm trocar. The main operating port site during transverse colectomy and right or extended right colectomy was placed in the left upper abdomen; however, during left colectomy it was placed in the right upper abdomen. In both cases, a 12-mm trocar was used.

#### Laparoscopic transverse colectomy

**Figure 1:**
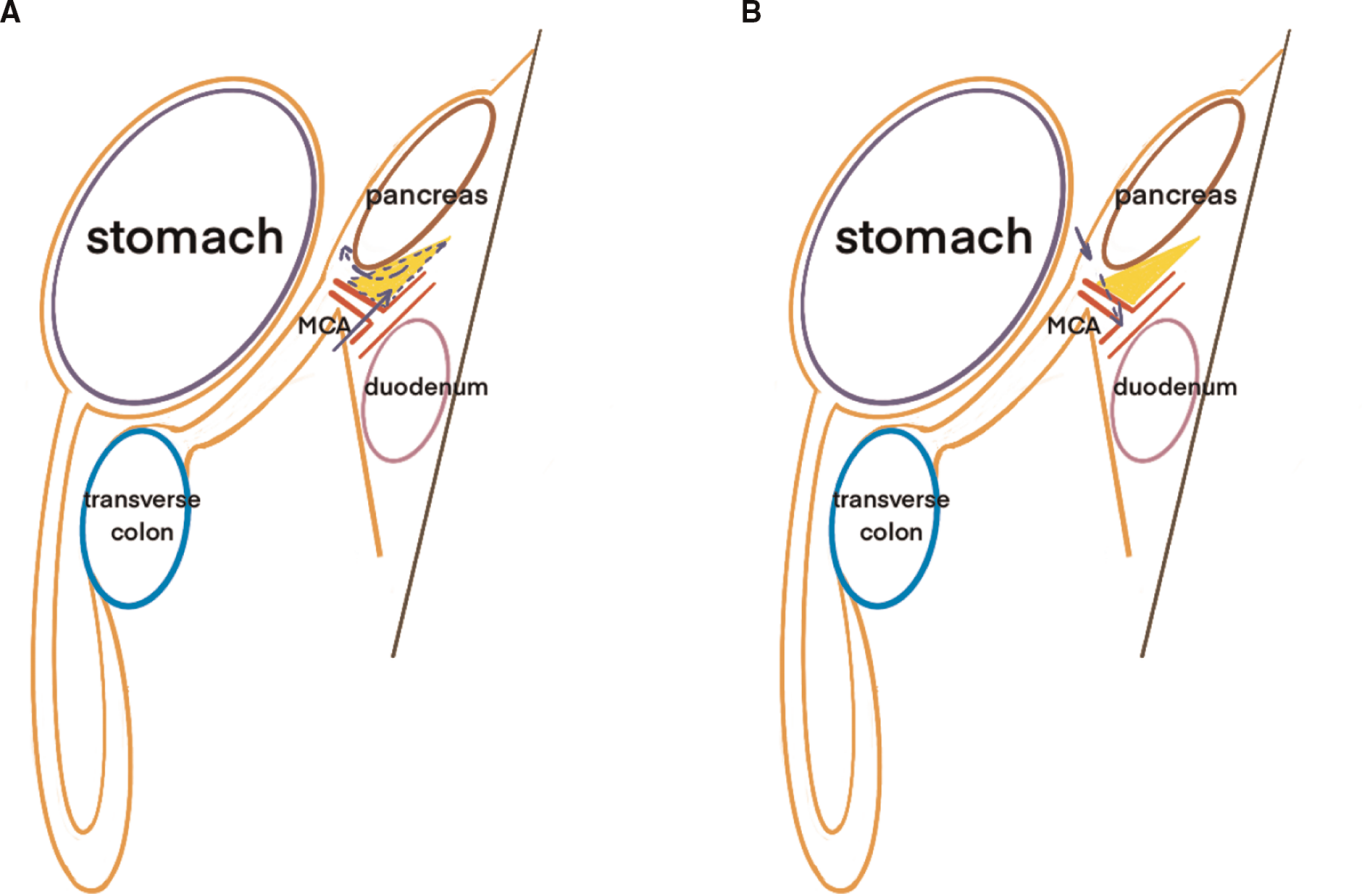
Schematic diagram of the different approaches in transverse colectomy. Note: (**A**) was referred to the caudal-to-cephalic approach and (**B**) was referred to the caphalic-to-caudal approach. The yellow triangle was referred to the lymph nodes concealed beneath and behind the lower border of pancreas which could be missed in cephalic-to-caudal approach. The arrow was referred to the different surgical routes.

Laparoscopic transverse colectomy is defined as surgery involving ligation of the MCA at its root while preserving the ileocolic (ICA) and left colic arteries (LCA). At least 10 cm of normal bowel surrounding the lesion were removed and the D3 lymphadenectomy was performed. D3 lymphadenectomy involves the dissection of the pericolic and intermediate lymph nodes up to the main lymph nodes at the origin of the MCA (223), as defined by the Japanese Society for Cancer of the Colon and Rectum (13). Surgery was performed using caudal-to-cephalic or cephalic-to-caudal approaches. And in both approaches, the extension of lymphadenectomy was alway D3. The caudal-to-cephalic approach involves pulling the transverse colon and the attached greater omentum towards the upper abdomen to adequately expose the base of the transverse mesocolon. Surgeons then locate the projection of the MCA at the conjunction of the base of the transverse mesocolon, ascending mesocolon, and Treitz ligament. The MCA and middle colic vein (MCV) are ligated in the root and the main lymph nodes (223) are removed. Subsequent lymphadenectomy is performed at the lower pancreatic border along the embryological plane. Lymphatic adipose tissue between the lower border of the pancreatic head and the transverse mesocolon is also carefully removed. The cephalic-to-caudal approach is initiated by dissecting the greater omentum and entering the lesser omental sac to separate the transverse mesocolon, which is then dissected from the lower pancreatic border. The MCA and MCV are detached and divided, and the lymph nodes are dissected at the origin of the main trunk artery. The anatomical schematic diagram of the two approaches was shown in Figure 1. In transverse colectomy, the gastrocolic trunk (GCT) and right colic artery (RCA) are not separated specifically.

The hepatic and/or splenic flexures are mobilized to ensure tension-free anastomosis. Anastomosis is performed either intracorporeally or extracorporeally, wherein intracorporeal anastomosis is performed in a side-to-side configuration, and extracorporeal anastomosis is performed using either a side-to-side or side-to-end configuration.

#### Laparoscopic hemicolectomy

Laparoscopic right hemicolectomy involves ligation of the ICA and right branch of the MCA, whereas laparoscopic extended right hemicolectomy involves ligation of the ICA and root of the MCA. Similarly, laparoscopic left hemicolectomy involves ligation of the LCA and the left branch of the MCA, whereas laparoscopic extended left hemicolectomy involves ligation of the LCA and the root of the MCA. The decision to ligate one branch of the MCA or the root of the MCA depends on the relative position of the tumor in regards to the feeding artery, according to the Japanese Classification of Colorectal, Appendiceal, and Anal Carcinomas (14). The bowel is resected from the terminal ileum to 10 cm distally to the lesion in right hemicolectomy or from the conjunction of the descending and sigmoid colon to 10 cm proximally to the lesion. During hemicolectomy, sharp dissection along the anatomical plane is necessary, and lymphadenectomy may extend to D2 or D3 levels according to tumor staging or patient status. In D3 surgery, the GCT and RCA was separated and ligated in their roots. The main lymph nodes at the root of the MCA (223) are always dissected during both D2 and D3 surgeries. Anastomosis is performed extracorporeally or intracorporeally and both side-to-side and side-to-end configurations are used.

### Outcomes

3-year DFS is the primary outcome of this study. Disease-free survival (DFS) is defined as the time from surgery to recurrence or metastasis, as confirmed by objective examination, or death due to any cause.

Secondary outcomes including 5-year overall survival (OS), the incidence of overall postoperative complications and postoperative hospital stay. OS is defined as the time from surgery to death from any cause. Postoperative complications are defined as adverse events that occurred within 30 days following surgery. The complications are classified according to Clavien-Dindo grading (15). Discharge criteria included absence of complications and tolerance to liquid diet. Administration of adjuvant therapy and follow-up strategy were previously described (16).

### Questionnaire investigation

Functional outcomes of the enrolled patients were investigated using a comprehensive online questionnaire or by telephone. The questionnaire was composed of the Bristol Stool Scale score and other questions that focused on the impact of defecation on quality of life, as previously documented (10, 17).

### Data analysis

Clinical data were collected from a prospectively established database. Complications that occurred after discharge were identified and registered by administrators. Clinical data, including baseline characteristics, operation information, recovery data, complications, and pathological parameters other than functional data of enrolled patients were retrospectively extracted and confirmed by electronic medical records. Categorical parameters were compared using the chi-square or Fisher exact test, and continuous parameters were compared using the Student *t*-test. The Kaplan–Meier method and log-rank test were used to compare DFS and OS rates. Statistical significance was set at *P*-values of <0.05. Data analyses were conducted using R software (version 4.0.3, R Foundation for Statistical Computing, Vienna, Austria, 2020, https://www.R-project.org).

## Results

**Figure 2:**
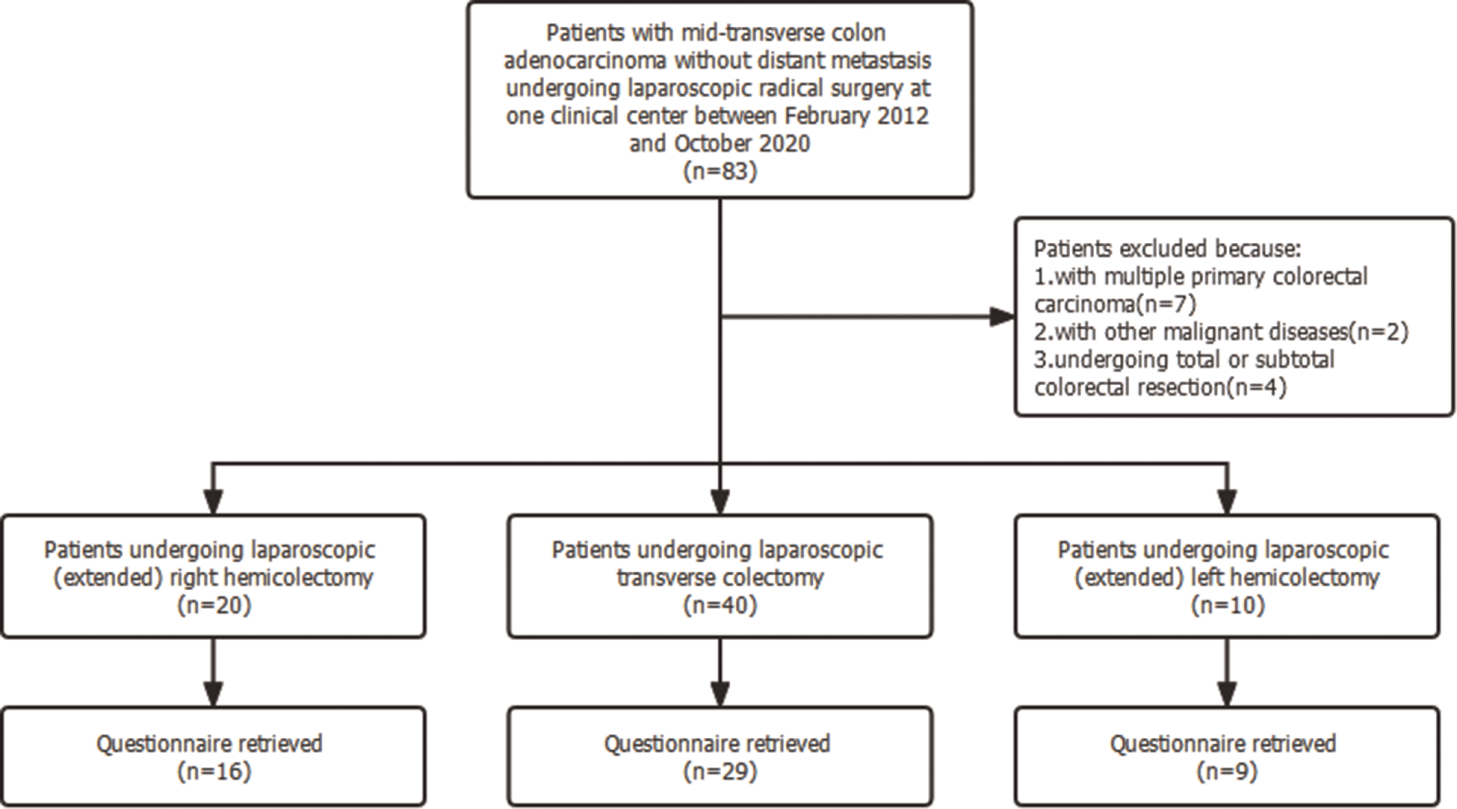
Flow diagram of the study.

Seventy patients fulfilled the recruitment criteria and were included in the study (Figure 2). Among them, 40 patients underwent laparoscopic transverse colectomy, 20 patients (extended) right hemicolectomy, and 10 patients (extended) left hemicolectomy. The baseline clinical and pathological characteristics of patients undergoing laparoscopic transverse colectomy were comparable to those of patients undergoing laparoscopic hemicolectomy (Table 1).

**Table 1:** Baseline characteristics.

Item	Laparoscopic transverse colectomy (*n* = 40)	Laparoscopic hemicolectomy (*n* = 30)	*P* value
Age, y	58.5 ± 12.5	61.9 ± 12.3	0.264
Male, *n* (%)	15 (37.5)	17 (56.7)	0.111
BMI, kg/m^2^	24.2 ± 2.9	24.9 ± 2.6	0.264
ASA, *n* (%)			0.584
I	4 (10.0)	5 (16.7)	
II	24 (60.0)	15 (50.0)	
III	12 (30.0)	10 (33.3)	
pT, *n* (%)			0.499
T_is_	1 (2.5)	0 (0.0)	
T1	1 (2.5)	0 (0.0)	
T2	4 (10.0)	2 (6.7)	
T3	30 (75.0)	21 (70.0)	
T4	4 (10.0)	7 (23.3)	
pN, *n* (%)			0.652
N0	23 (57.5)	20 (66.7)	
N1	10 (25.0)	7 (23.3)	
N2	7 (17.5)	3 (10.0)	
AJCC stage, *n* (%)			0.769
TisN0	1	0	
I	3	2	
II	19	18	
III	17	10	
Differentiation degree, *n* (%)			0.181
Well and moderate	27 (67.5)	25 (83.3)	
Poor	6 (15.0)	4 (13.3)	
Mucinous adenocarcinoma	7 (17.5)	1 (3.3)	
Vascular or lymphatic invasion, *n* (%)			0.819
Present	11 (27.5)	9 (30.0)	
Absent	29 (72.5)	21 (70.0)	
Perineural invasion, *n* (%)			1.000
Present	3 (7.5)	3 (10.0)	
Absent	37 (92.5)	27 (90.0)	

**Figure 3:**
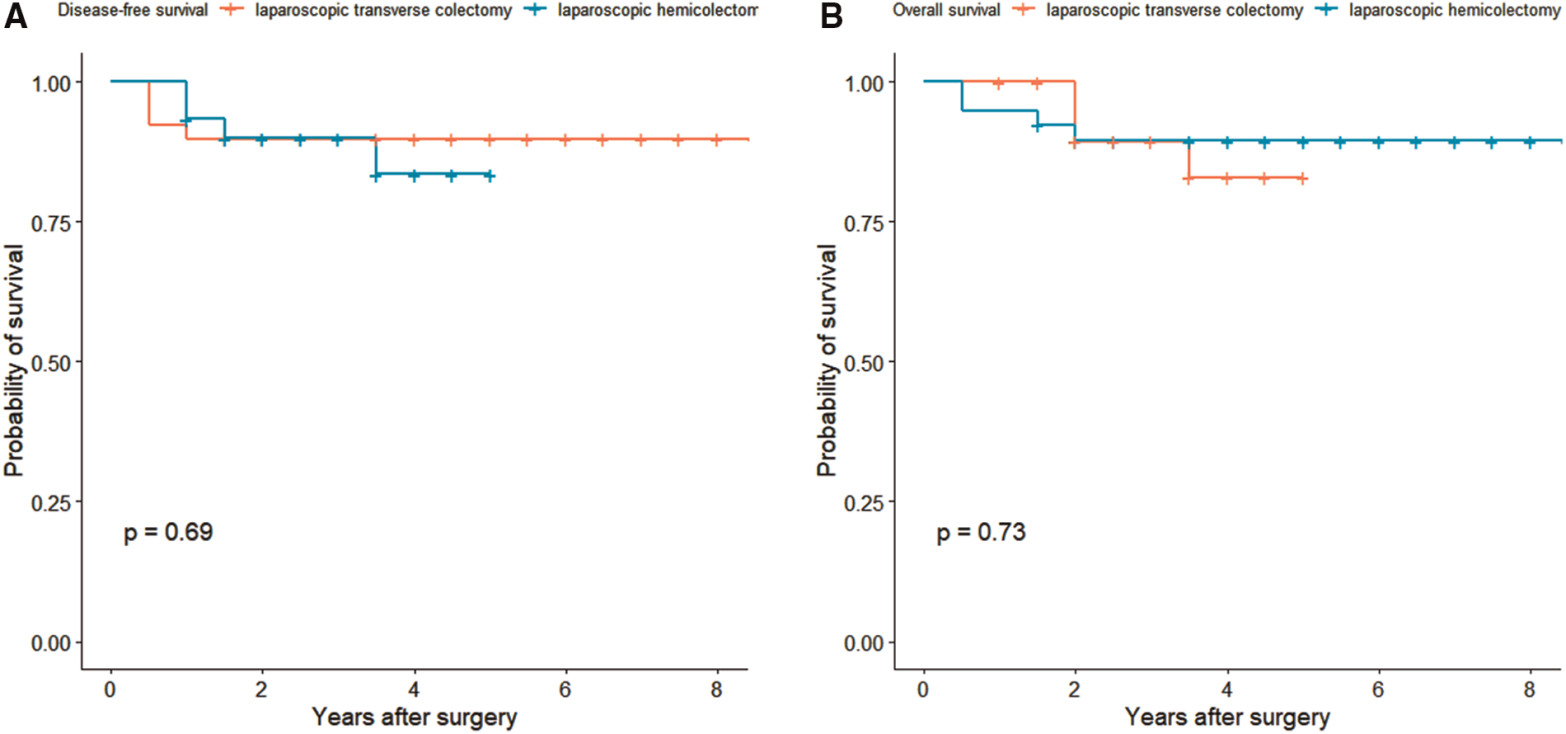
(**A**) Disease-free survival of the two groups. (**B**) Overall survival of the two groups.

The median follow-up time was 3.5 years (interquartile range 2–5 years). One patient in the laparoscopic transverse cohort was lost to follow up. The 3-year disease-free survival rates in the laparoscopic transverse colectomy and hemicolectomy cohorts were 89.7% (95% CI 80.7%–99.8%) and 89.9% (95% CI 79.6%–100.0%), respectively (*P* = 0.688) (Figure 3A). The 5-year overall survival rate was 89.4% (95% CI 80.1%–99.8%) in laparoscopic transverse colectomy cohort and 82.9% (95% CI 68.3%–100.0%) in the laparoscopic hemicolectomy cohort (*P* = 0.726) (Figure 3B). The overall incidence of postoperative complications was lower in the laparoscopic transverse colectomy cohort than in the laparoscopic hemicolectomy cohort although without statistical significant (8/40 [20.0%] vs. 12/30 [40.0%], *P* = 0.067). The postoperative hospital stay was shorter in the laparoscopic transverse colectomy cohort than in the laparoscopic hemicolectomy cohort also without statistical significance (7 [5–12] vs. 7 [5–18], *P* = 0.060) (Table 2).

**Table 2:** Operative details and postoperative short-term outcomes.

Item		Laparoscopic transverse colectomy (*n* = 40)	Laparoscopic hemicolectomy (*n* = 30)	*P* value
Operation time, min		171.0 ± 43.3	163.0 ± 40.9	0.436
Estimated blood loss, ml		53.0 ± 50.8	61.2 ± 56.1	0.527
Mobilization of colonic flexures, *n* (%)				–
Hepatic flexure		19 (47.5)	–	
Splenic flexure		8 (20.0)	–	
Both flexures		6 (15.0)	–	
Neither flexures		7 (17.5)	–	
Intraoperative complications, *n* (%)				
Multiple organ resection		3 (7.5)	1 (3.3)	0.630
Vascular injury		1 (2.5)	0 (0.0)	1.000
Conversion to open surgery, *n* (%)		0 (0.0)	1 (3.3)	0.429
Conversion to hemicolectomy, *n* (%)		–	3	–
Number of lymph nodes harvested		19.7 ± 12.8	27.4 ± 15.3	0.025
Ambulation time, days^a^		1 (1–4)	1 (1–2)	0.383
Time to first flatus, days^a^		2 (1–4)	2 (1–4)	0.669
Time to fluid diet, days^a^		5 (4–7)	5 (3–12)	0.715
Postoperative hospital stay, days^a^		7 (5–12)	7 (5–18)	0.060
Postoperative complications, *n* (%)^b^		8 (20.0)	12 (40.0)	0.067
Clavein-Dindo Grade I	Diarrhea	1	1	
	Chylous leak	1	1	
	Wound infection	1	1	
Clavein-Dindo Grade II	Pneumonia	1	1	
	Pulmonary embolism	1	0	
	Abdominal infection	0	1	
	Urinary infection	1	1	
	Diarrhea	1	2	
	paralytic ileus	0	4	
	Chylous leak	0	1	
Clavein-Dindo Grade III	Anastomotic leak	0	1	
	Intestinal internal hernia	1	0	
	paralytic ileus	0	1	
	Wound infection	1	0	

^a^ Data was shown by median with range.

^b^ Patients could have more than one kind of complication. This parameter was referred to the number of patients who were affected by complications. The detailed complications below were referred to number of events diagnosed.

The operative details of the two groups are shown in Table 2. Among the transverse colectomy surgery cohort, mobilization of the colic splenic and/or hepatic flexure was needed in 33 (82.5%) cases. Meanwhile, extended hemicolectomy was required in 19 (63.3%) cases of the hemicolectomy cohort. The mean operating time of both groups did not differ significantly (*P* = 0.463), nor did the mean estimated blood loss (*P* = 0.527). However, the mean number of harvested lymph nodes was significantly lower in the transverse colectomy cohort than in the hemicolectomy cohort (19.7 ± 12.3 and 27.4 ± 15.3, *P* = 0.025). Twenty-six (65.0%) patients underwent transverse colectomy using the caudal-to-cephalic approach, while 14 (35.0%) underwent the cephalic-to-caudal approach. The mean operating time was significantly longer and the mean number of harvested lymph nodes was significantly higher in caudal-to-cephalic approach (operating time: 184.3 ± 37.1 vs. 146.3 ± 44.4, *P* = 0.012; total harvested lymph nodes: 23.1 ± 14.3 vs. 13.4 ± 5.4, *P* = 0.004) (Table 3).

**Table 3:** Comparison of different approaches of laparoscopic transverse colectomy.

Item	Caudal approach (*n* = 26)	Cephalic approach (*n* = 14)	*P* value
Operative time, min	184.3 ± 37.1	146.3 ± 44.4	0.012
Estimated blood loss, ml	48.7 ± 42.0	61.1 ± 65.0	0.526
Number of lymph nodes harvested	23.1 ± 14.3	13.4 ± 5.4	0.004

**Table 4:** The incidences of bowel dysfunction in the laparoscopic transverse colectomy and hemicolectomy.

Outcome	Laparoscopic transverse colectomy (*n* = 29)	Laparoscopic hemicolectomy (*n* = 25)	*P* value
Bristol stool scale score 6–7, *n* (%)	1 (3.4)	3 (12.0)	0.326
Four or more bowel movements daily, *n* (%)	2 (6.9)	2 (8.0)	1.000
Bowel function impact on QoL, *n* (%)	13 (44.8)	6 (24.0)	0.113
Alternating consistency, *n* (%)	15 (51.7)	20 (80.0)	0.030
More than 5 min per attempt to defecate,^b^ *n* (%)	13 (44.8)	7 (28.0)	0.202
Urgency^a^, *n* (%)	3 (10.3)	2 (8.0)	1.000
Unproductive call to defecate^a^, *n* (%)	6 (20.7)	1 (4.0)	0.108
Strain to defecate, *n* (%)	4 (13.8)	2 (8.0)	0.675
Obstructive sensation^a^, *n* (%)	0 (0.0)	1 (4.0)	0.463
Incomplete evacuation^a^, *n* (%)	3 (10.3)	3 (12.0)	1.000
Clustering^a^, *n* (%)	2 (6.9)	5 (2.0)	0.229
Nocturnal bowel movement^a^, *n* (%)	1 (3.4)	2 (8.0)	0.591
Flatulence^a^, *n* (%)	5 (17.2)	1 (4.0)	0.200
Ability to defer defecation for more than 15 min, *n* (%)	22 (75.9)	19 (76.0)	0.991
Incontinence flatus^a^, *n* (%)	2 (6.9)	1 (4.0)	1.000
Incontinence liquid^a^, *n* (%)	0 (0.0)	1 (4.0)	0.463
Incontinence solid^a^, *n* (%)	3 (10.3)	1 (4.0)	0.615
Use of pads^a^, *n* (%)	0 (0.0)	1 (4.0)	0.463
Soiling^a^, *n* (%)	0 (0.0)	0 (0.0)	1.000
Antidiarrheal agents,^b^ *n* (%)	3 (10.3)	4 (16.0)	0.692

^a^ Symptoms occuring at least once a week.

^b^ The symptom/treatment being present or used.

Among the postoperative complications, CD III complications did not differ between the two groups (Table 2). However, two patients in the laparoscopic transverse colectomy cohort experienced complications that required reoperation; one patient required release of an intestinal internal hernia, while the other underwent debridement due to severe wound infection. One patient in the laparoscopic hemicolectomy cohort had an anastomotic leak and subsequently underwent ostomy. One patient undergoing laparoscopic extended right hemicolectomy suffered from paralytic ileus which required the invasive ileus tube. Postoperative complications are shown in Table 2. Ambulation time, time to first flatus and time to fluid diet after surgery were comparable between both groups.

A questionnaire was distributed to all 70 patients to investigate functional outcomes: 29 (72.5%) patients in the laparoscopic transverse colectomy cohort and 25 (83.3%) patients in the hemicolectomy cohort completed the questionnaire (*P* = 0.285) (Figure 1). The mean time from surgery to the questionnaire was filled was 4.7 ± 2.0 years. The results showed that alternating consistency in defecation occurred significantly more frequently during laparoscopic hemicolectomy (*P* = 0.030). However, no significant differences in the Bristol Stool Scale score (6, 7) and daily bowel movements (≥4 per day) existed between the cohorts. The impact of bowel function on the quality of life and other symptoms included in the questionnaire was comparable in both groups (Table 4).

## Discussion

The outcomes and optimal surgical approaches of mid-transverse colon cancer remains not completely studied and more clinical evidence is expected. Our study noted that the D3 laparoscopic transverse colectomy was less invasive and associated with similar oncological and functional outcomes compared to those of laparoscopic hemicolectomy.

A previous study reported that transverse colectomies decreased over the past decade (6). This may be explained by the surgeons' hesitation to perform laparoscopic transverse colectomies since they cannot easily access the targeted embryological plane necessary to accomplish sharp dissection. However, whether the oncological outcomes of laparoscopic transverse colectomy allow its use as a surrogate to hemicolectomies remains debatable. In our cohort study, the mean operation time, estimated blood loss, and intraoperative complications of transverse colectomy were comparable to those of hemicolectomy. These results show that transverse colectomies can be used to resect mid-transverse colon cancers by experienced surgeons.

Complete mesocolic excision is of utmost importance when performing transverse colectomy to ensure favorable oncological outcomes. Transverse mesenteric lymph nodes are partially concealed in the fold below the pancreas. In the caudal-to-cephalic approach, retracting the transverse mesocolon cephalically and initiating the cutting line on the dorsal side of the mesocolon is more convenient and efficient to completely remove the mesenteric lymph nodes. While in the cephalic-to-caudal approach, surgeons have to cross over the pancreas to remove the lymph nodes folded beneath and behind the pancreas, which is more difficult to do lymphadenectomy completely. The cutting line was predetermined to be longer in the caudal-to-cephalic approach; therefore, as our results showed, a longer operative time was required. Since in our operations D3 lymphadenectomy was performed for all transverse colectomy, the difference of harvested lymph nodes between the two approaches was less likely to be associated with the lymph nodes surrounding the root of MCA than associated with the lymph nodes shaded by the pancreas. More lymph nodes harvested in the caudal-to-cephalic approach implied advanced lymphadenectomy, which may lead to better therapeutic outcomes.

The resected bowel length was not measured in fresh specimens in our study. The proximal and distal margins were accounted for when performing colectomies since resection of at least 10 cm bowel was regarded as a basic principle to remove sufficient pericolic lymph nodes (13). In our study, transverse colectomy also conformed to this basic principle, and we found that the resected margin could be guaranteed if the bowel was sufficiently mobilized. However, mobilization of the transverse colon as well as both flexures is technically challenging and should be performed meticulously to avoid accidental damage to the blood supply. In our study, two cases from the transverse colectomy cohort were subsequently converted to right colectomy due to poor blood perfusion to the proximal bowel.

Regarding short-term outcomes, transverse colectomy appeared to marginally decrease the risk of complications and the length of hospital stay, although the difference was not statistically significant. Overall, the CD grading distribution of complications and the risk of CD III complications were equivalent between both groups. A previous study showed that the incidence of postoperative complications did not differ between both groups (18). A recent meta-analysis also found transverse colectomy might be associated with less postoperative ileus (19). However, one study reported that transverse colectomy was associated with a significantly higher incidence of anastomotic leaks, anemia, and wound infections (9). But in this study, the open transverse colectomy accounted for 57.7% of all transverse colectomies, which could elevate the mortality after surgery. Meanwhile the recovery parameters namely time to first flatus, time to first mobilization, and overall hospital stay were all significantly shorter in the hemicolectomy group in this study, which is hard to interpret because the more invasive procedure on the contrary resulted in better outcomes. The laparoscopic transverse colectomy technology continues to advance. Our results suggest that a moderate dissection range in transverse colectomy may be beneficial to short-term outcomes of patients; nevertheless, this benefit should be cautiously considered based on comparable oncological outcomes.

To account for balanced demographic and pathological characteristics, we found that oncological outcomes, regardless of DFS or OS, were comparable between both groups. This result was comparable to the recent meta-analysis (19). Due to our relatively small sample size, the endpoint events were limited in our study; however, we found that oncological outcomes were not compromised in the transverse colectomy group.

The oncological outcomes of transverse colectomy emphasize the pattern of lymph node metastasis in mid-transverse colon cancer. Park et al. (20) reported no metastatic lymph nodes along the side of the ICA and LCA in mid-transverse colon cancer, indicating that resection of these lymph nodes might be redundant. Fukuoka et al. (7) also stated that invasion and metastasis of mid-transverse colon cancers mainly occurred through the MCA. However, other researchers reported lymph node metastasis along the side of the RCA, which may be missed when performing transverse colectomy (20). This may be influenced by the relative proximity of the tumor to the RCA. For an example, Fukuoka et al. reported that lymph node metastasis surrounding the RCA tends to occur on the right side of the transverse colon (7). Therefore, reasonable patient selection is important when performing transverse colectomy. Studies also found that the pathological stage was significantly earlier, and unfavorable pathological characteristics were less frequently in the transverse colectomy group (6, 7). Regardless of the surgical approach, the lymph node dissection along the side of the MCA, especially 223 lymph nodes, is crucial in treating the disease.

Few studies have discussed the long-term functional outcomes of transverse colectomy vs. hemicolectomy. Some studies suggest that laparoscopic right hemicolectomy is associated with chronic diarrhea due to ileocecal valve and terminal ileum resection (21, 22). However, our study reported equivalent Bristol stool score and bowel movements per day between both cohorts. We found that alternating consistency of defecation occurred more frequently after laparoscopic hemicolectomy, but this gap was too narrow to present any clinical-relevant difference. The hemicolectomy group included left hemicolectomy. This might possibly elevate the functional presentations of this group since left hemicolectomy also invovles colo-colonic anastomosis.

This study had several limitations that need to be addressed. First, we enrolled a relatively small sample size, which affected the statistical power of our results. Second, the surgical choice was made according to the experience of different surgeons and no uniform standard was followed, which might lead to potential imbalance of the baseline characteristics. Finally, baseline functional characteristics were lacking, which may have influenced interpretation of the functional results.

## Conclusion

In short, laparoscopic transverse colectomy is a technically feasible, safe, and satisfactory alternative for treating mid-transverse colon cancer. To ensure optimal oncological outcomes, laparoscopic transverse colectomy is recommended for early stage mid-transverse colon cancer. In addition, the caudal-to-cephalic approach is more advantageous than alternatives in accomplishing complete lymphadenectomy.

## Data Availability

The raw data supporting the conclusions of this article will be made available by the authors, without undue reservation.
